# Gossip, sabotage, and friendship network dataset

**DOI:** 10.1016/j.dib.2021.107717

**Published:** 2021-12-16

**Authors:** Meltem Yucel, Gustav R. Sjobeck, Rebecca Glass, Joshua Rottman

**Affiliations:** a Department of Psychology, University of Virginia, United States; b Department of Psychology and Neuroscience, Duke University, United States; c Widener University, Institute for Graduate Clinical Psychology (IGCP), United States; d Franklin and Marshall College, Psychology and Scientific and Philosophical Studies of Mind, United States

**Keywords:** Friendship, Gossip, Negative gossip, Positive gossip, Sex gossip, Sabotage, Social network analysis

## Abstract

This article describes the data reported in the paper “Being in the know: Social network analysis of gossip and friendship on college campuses” (Yucel et al. 2021). Data were collected from a Men's and Women's collegiate crew team members from a small liberal arts college. Participants (*N* = 44) reported information about how often they gossip about members of the team (positively, negatively), who they have had hooked-up with on the team, who they consider to be friends with on the team, whether they have to sabotaged or been sabotaged by any teammates, their well-being and feelings of loneliness. This data brief provides detailed information about data preparation and participants responses to all survey items.

**Specifications Table**

**Table:** 

Subject	Psychology (General)
Specific subject area	Gossip and Friendship Social Network Analysis
Type of data	Table
How data were acquired	Data were acquired online, using Qualtrics. Gossip networks (positive, negative, hook-up, and sabotage), friendship network, General Well-Being Schedule [2] and the R-UCLA Loneliness Scale [3] were acquired using these surveys.
Data format	Raw (anonymized): “Gossip_and_Well-Being-RAW.csv” (also for SPSS “Gossip_and_Well-Being-RAW.sav”) Analyzed (anonymized): “Gossip_and_Well-Being-ANALYZED.csv” (also for SPSS “Gossip_and_Well-Being-ANALYZED.sav”)
Parameters for data collection	Participants were members of a Men's and Women's collegiate crew team. Participants were all over the age of 18.
Description of data collection	Data were acquired online, using two Qualtrics surveys. The first survey collected participant names and consents. The second survey asked participants to list how often they gossip about members of the team (positively, negatively), who they have had hooked-up with on the team, who they consider to be friends with on the team, whether they have to sabotaged or been sabotaged by any teammates, General Well-Being Schedule [2] and the R-UCLA Loneliness Scale [3].
Data source location	Institution: Franklin & Marshall College City/Town/Region: Lancaster, PA Country: USA
Data accessibility	Our data can be found on Mendeley Data https://data.mendeley.com/datasets/kpjjvg39k3
Related research article	Yucel, M., Sjobeck, G. R., Glass, R., & Rottman, J. (2021). Being in the know: Social network analysis of gossip and friendship on a college campus. *Human Nature.*10.1007/s12110-021-09409-5

**Value of the Data**

• To date, no data distinguishing different types of gossip behaviors in a closed network exists.
• These data will enable researchers to create comprehensive gossip surveys that can be used at larger social networks.
• Researchers can explore different personal (e.g., feelings of loneliness) or social factors (e.g., sabotage behavior) that might influence social network structures.

## 1 Data Description

The anonymized raw data file (i.e., “Gossip_and_Well-Being-RAW.csv”) includes participant ID number, gender, responses to General Well-Being Schedule [2], the R-UCLA Loneliness Scale [3], reports of negative gossip about others (NegGos), positive gossip about others (PosGos), hook-up gossip about others (HUGos), whether or not they have hooked-up with others on the team (HU), who they consider to be friends with on the team (Friends), and whether they have sabotaged (DoneSab), or been sabotaged by any teammates (BeenSab).

The anonymized analyzed data file (i.e., “Gossip_and_Well-Being-ANALYZED.csv”) includes participant ID number, gender, overall General Well-Being Schedule [2] score, overall the R-UCLA Loneliness Scale [3] score, reports of negative gossip about others (NegGos), positive gossip about others (PosGos), hook-up gossip about others (HUGos), whether or not they have hooked-up with others on the team (HU), who they consider to be friends with on the team (Friends), and whether they have sabotaged (DoneSab), or been sabotaged by any teammates (BeenSab). The file also includes variables for incoming positive gossip (PosGosIN), incoming negative gossip (NegGosIN), incoming hook-up gossip (HUGosIN), incoming friendship (FriendsIN), outgoing positive gossip (PosGosOUT), outgoing negative gossip (NegGosOUT), outgoing hook-up gossip (HUGosOUT), and outgoing friendship (FriendsOUT) (Table 1).

**Table 1:** Descriptive information of participants responses to surveys

Measures	*N*	Min.	Max.	*M (SD)*
RUCLA	44	33	51	39.82 (4.61)
GWBS	44	54	122	90.68 (15.52)
Number of sexual partners had at College	44	0	25	3.14 (4.97)
Incoming Positive Gossip	44	0	43	14.91 (8.84)
Incoming Negative Gossip	44	0	14	6.84 (3.97)
Incoming Hook-up Gossip	44	0	15	4.14 (3.89)
Incoming Friendship	44	9	41	23.32 (7.27)
Outgoing Positive Gossip	44	3	30	14.91 (6.06)
Outgoing Negative Gossip	44	0	21	6.84 (5.38)
Outgoing Hook-up Gossip	44	0	18	4.14 (4.72)
Outgoing Friendship	44	7	33	23.32 (5.96)
Have Sabotaged	44	0	0.07	0.00 (0.01)
Been Sabotaged	44	0	0.05	0.00 (0.01)

## 2 Experimental design, materials and methods

Participants on the Men's and Women's crew team were first approached to give consent to be involved in the study. All but one crew team members gave permission to be included in the study [1]. A roster was created with team members who gave consent. Participants were then sent an online survey asking them how often they positively gossip about members of the team, negatively gossip about members of the team, spread hook-up gossip about members of the team, who they have had hooked-up with on the team, who they consider to be friends with on the team, whether they have sabotaged or been sabotaged by any teammates, General Well-Being Schedule [2], and the R-UCLA Loneliness Scale [3], and how many hook-up partners they have had at the university.

### 2.1 Materials

*General Well-Being Schedule.* The General Well-Being Schedule [2] was used to identify a subject's wellbeing and possible distress. The scale measures happiness, life satisfaction, emotional stability, restedness, nervousness, anxiety, stress, and pressure a participant may be experiencing in the past month (e.g., “Have you been in firm control of your behavior, thoughts, emotions OR feelings? (During the past month)”). It is a 25-item survey with responses assessed on a Likert scale that varied in range and anchors. High scores are indicative of positive adjustment and higher well-being, the highest score being 110 and lowest score being 0, indicating severe distress.

*R-UCLA Loneliness Scale.* The R-UCLA Loneliness Scale [3], which is a 20-item measure, was used to identify the relationship between loneliness, social relationships, and emotional states. It is also used to determine whether a participant feels connected/part of a group (e.g., “I feel part of a group of friends,” “My interests and ideas are not shared by those around me”). All Likert scale responses range from Never (1) to Often (4). The highest possible score is an 80, indicating a higher amount of loneliness and lowest possible score of 20, indicating less loneliness.

*Reports of negative gossip about others (NegGos).* A roster was created containing the names of individuals on both the men's and women's crew teams. Participants were asked about how often they gossiped negatively about each member on the team (with responses ranging from 1, Never, to 7, Daily) (e.g., “How much do you tend to spread negative gossip about each of the following individuals?”). This item was written specifically for this data collection.

*Reports of positive gossip about others (PosGos).* A roster was created containing the names of individuals on both the men's and women's crew teams. Participants were asked about how often they gossiped positively about each member on the team (with responses ranging from 1, Never, to 7, Daily) (e.g., “How much do you tend to spread positive gossip about each of the following individuals?”).. This item was written specifically for this data collection.

*Reports of hook-up gossip about others (HUGos).* A roster was created containing the names of individuals on both the men's and women's crew teams. Participants were asked about how often they spread hook-up related gossip about each member on the team (with responses ranging from 1, Never, to 7, Daily) (e.g., “How much do you tend to spread hook-up related gossip about each of the following individuals?”). This item was written specifically for this data collection.

*Reports of whether they have hooked-up with others on the team (HU).* A roster was created containing the names of individuals on both the men's and women's crew teams. Participants were asked a simple Yes–No question about whether they have ever hooked up with any of the team members (e.g., “Have you ever hooked up with any of the following individuals? (Remember that all responses are anonymized)”). This item was written specifically for this data collection.

*Reports of who they consider to be friends with on the team (Friends).* A roster was created containing the names of individuals on both the men's and women's crew teams. Participants were asked a simple Yes–No question about whether they considered each team member to be a friend (e.g., “Do you consider yourself to be friends with the following individuals?”). This item was written specifically for this data collection.

*Reports of whether they have sabotaged any teammates (DoneSab).* A roster was created containing the names of individuals on both the men's and women's crew teams. Participants were asked a simple Yes–No question about whether they have ever tried to sabotage any of the team members (e.g., “Have you ever tried to sabotage any of the following individuals?”). This item was written specifically for this data collection.

*Reports of whether they have been sabotaged by any teammates (BeenSab).* A roster was created containing the names of individuals on both the men's and women's crew teams. Participants were asked a simple Yes–No question about whether they have ever been sabotaged by any of the team members (e.g., “Have you ever been sabotaged by any of the following individuals?”). This item was written specifically for this data collection.

### 2.2 Methods

*Data cleaning.* The raw data file was anonymized to protect the identity of the participants and is shared as “Gossip_and_Well-Being-RAW.csv” (also for SPSS “Gossip_and_Well-Being-RAW.sav”). Raw data was analyzed using “Gossip_and_Well-Being-SYNTAX.sps” (for text file version “Gossip_and_Well-Being-SYNTAX.rtf”. The Analyzed data is shared as “Gossip_and_Well-Being-ANALYZED.csv” (also for SPSS “Gossip_and_Well-Being-ANALYZED.sav”). The Analyzed data file has been expanded upon to include the following information: GWBS items 1 to 16, and items 24 and 25 were reverse coded. An overall GWBS score was derived by summing all GWBS items. RUCLA items 1, 4, 5, 6, 9, 10, 15, 16, 19, and 20 were reverse coded. An overall RUCLA score was derived by summing all RUCLA items. All negative gossip, positive gossip, hook-up gossip, hook-up, have sabotaged others, been sabotaged, and friendship responses have also been recoded as a binary (Yes/No) response. In addition, incoming and outgoing network information were coded into separate variables.

*In degree and out degree.* In degree and out degree were calculated using the centrality function in R, from the qgraph package [4]. In degree is a measure of the number of edges in the network that point towards a given node, whereas out degree is a measure of the number of edges in the network that point away from a given node. Similarly, eigenvector centrality was calculated in R using the eigen_centrality function, which can be found in the igraph package [5]. This function measures the prestige of a given node, where nodes with high prestige are those that are well-connected to other well-connected nodes. All three measures are considered to reflect the centrality of a node in a network.

## Ethics Statement

The questionnaire and methodology for this study was approved by the Human Research Ethics committee of the Franklin & Marshall College, on 02/07/2017 for the application #R_z8tMp5lSWgH7STL. All participants provided consent to be involved in the study.

## Declaration of Competing Interest

This work was supported by Psi Chi, The International Honor Society in Psychology and the Franklin & Marshall College Committee on Grants. The authors declare that they have no known competing financial interests or personal relationships which have, or could be perceived to have, influenced the work reported in this article.
